# Coupling Dynamic Covalent Bonds and Ionic Crosslinking Network to Promote Shape Memory Properties of Ethylene-vinyl Acetate Copolymers

**DOI:** 10.3390/polym12040983

**Published:** 2020-04-23

**Authors:** Wenjing Wu, Sreeni Narayana Kurup, Christopher Ellingford, Jie Li, Chaoying Wan

**Affiliations:** 1 International Institute for Nanocomposites Manufacturing (IINM), WMG, University of Warwick, Coventry CV4 7AL, UK; wuwenjing_2007@163.com (W.W.); S.Narayana-Kurup@warwick.ac.uk (S.N.K.); C.Ellingford@warwick.ac.uk (C.E.); 2 Aerospace Research Institute of Materials & Processing Technology, Beijing 100076, China; lijie@arimt.com

**Keywords:** ethylene-vinyl acetate copolymer, shape memory, reversible crosslinking, Diels–Alder reaction, ionic interactions

## Abstract

Dynamic crosslinking networks based on Diels–Alder (DA) chemistry and ionic interactions were introduced to maleic anhydride modified ethylene-vinyl acetate copolymer (mEVA) via in situ melt processing. The dual dynamic crosslinking networks were characterized by temperature-dependent FTIR, and the effects on the shape memory properties of mEVA were evaluated with dynamic mechanical thermal analysis and cyclic tensile testing. A crosslinking density was achieved at 2.36 × 10^−4^ mol·cm^−3^ for DA-crosslinked mEVA; as a result, the stress at 100% extension was increased from 3.8 to 5.6 MPa, and tensile strength and elongation at break were kept as high as 30.3 MPa and 486%, respectively. The further introduction of 10 wt % zinc methacrylate increased the dynamic crosslinking density to 3.74 × 10^−4^ mol·cm^−3^ and the stress at 100% extension to 9.0 MPa, while providing a tensile strength of 28.4 MPa and strain at break of 308%. The combination of reversible DA covalent crosslinking and ionic network in mEVA enabled a fixing ratio of 76.4% and recovery ratio of 99.4%, exhibiting an enhanced shape memory performance, especially at higher temperatures. The enhanced shape memory and mechanical performance of the dual crosslinked mEVA showed promising reprocessing and recycling abilities of the end-of-life products in comparison to traditional peroxide initiated covalent crosslinked counterparts.

## 1. Introduction

The shape memory effect is a phenomenon related to a physical state change of a polymer upon application of a stimulus. Heat-shrinkable shape memory products are extensively used in industries for electrical insulation, environmental protection, and wire bundling. For example, covalently crosslinked polyethylene has been used as heat-shrinkable tubing and cable sheathing [1]. In addition, polymers and composites with shape memory abilities can be used to develop smart energy-harvesting devices [2] and have become very useful for space and biomedical applications [3]. When the polymer is deformed under an external force, the temporary deformed shape is “frozen” whilst the permanent shape is memorized. Upon exposure to a trigger stimulus, such as temperature, light, electrical or magnetic field, pH, or solvent [4], a conformation transition is induced and the polymer recovers the memorized permanent shape. As an entropy-driven transformation, the conformation of polymer changes with the movement of internal molecular unit motion [5]. In the process of shape memory, there are two competing stages: the chain deformation under external stress and the chain contraction as an entropic response. To promote shape memory effect, a dual segment polymer system is commonly suggested. One segment is elastic to provide recovery ability; this is typically a polymer containing chemically or physically cross-linked structure, such as ionic bonds, π–π interactions, hydrogen bonding, or metal–ligand coordination [6,7]. The second segment fixes the temporary conformation by freezing the temporary shape in place. This is usually achieved through an amorphous/semicrystalline phase transition or intermolecular interactions. A combinatorial approach utilizes reversible bonds or supramolecular interactions to improve the shape memory performance of polymers, such as Diels–Alder bonds, disulfide bonds, and ionic interactions [8,9,10]. The reversibility of the dynamic bonds enables the transition between the shape deformation and shape fixing, through debonding at high temperatures and rebonding at low temperatures. The debonding at high temperatures during the second heating stage benefits the shape recovery by “unfreezing” the restrained polymer chains.

Ethylene-vinyl acetate copolymer (EVA) is a saturated linear polymer commonly used in automotive and electrical insulation applications, such as gaskets, hot melt adhesives, cable insulants, and shoe soles [11]. When used in cable insulants, it is usually subjected to various tensile or bending deformations under external conditions and suffers from aging and mechanical damage. Endowing the cable insulants with shape memory and shape recovery abilities can simplify the cable manufacturing process.

Melting transitions are often used to initiate the shape memory behavior of chemically crosslinked semicrystalline thermoplastics such as EVA, polyethylene, or polyesters. The polymer deforms when subjected to a temperature above its melting point [12,13,14]. This also enables the potential recycling or reprocessing of those chemically crosslinked polymers at the end of the service life. The introduction of multiple reversible crosslinking networks to EVA will potentially improve the shape memory and mechanical properties while retaining a good reprocessing ability of the polymer. The vinyl acetate unit in EVA provides polarity and elasticity but is not sufficient to recover the mechanical deformation. In this study, to keep the original shape-fixing ability of EVA through the melt–crystalline transition, reversible dynamic crosslinking networks were introduced into maleic anhydride modified EVA (mEVA). The reversible covalent bonds were firstly introduced to mEVA via Diels–Alder interactions [10,11], followed by ionic interactions through the addition of zinc dimethacrylate [15,16]. The aggregation of the ionic clusters acts as physical crosslinking sites responsible for enhanced flexibility, toughness, temperature sensitivity, and potential recovery function [6,7,17,18,19]. The effects of the reversible DA bonds and the ionic crosslinks on the mechanical and shape memory performance of modified mEVA were discussed.

## 2. Experimental Section

### 2.1. Materials

Maleic anhydride modified ethylene-vinyl acetate copolymer (mEVA, Fusabond C250) containing 2 wt % maleic anhydride and with a melt flow rate of 1.4 g/10 min (190 °C, 2.16 kg) was kindly provided by Dow Ltd. Furfurylamine (FFA, Sigma-Aldrich, ≥99%, Schnelldorf, Germany) and 4,4’-methylenebis(N-phenylmaleimide) (BM, Alfa Aesar, 95%, Heysham, UK) were used for the Diels–Alder reaction. Zinc methacrylate (ZnMA) and dicumyl peroxide (DCP, 98%) were purchased from Sigma-Aldrich.

### 2.2. Sample Preparation

To introduce DA bonds to mEVA, mEVA was melted in the mixing chamber at 140 °C for 1 min at a rotor speed of 60 rpm in a Haake rheometer, followed by the addition of FFA. After 15 min, BM was added and mixed for a further 10 min to produce mEVA-DA. Based on the maleic anhydride content, the theoretical concentrations of FFA and BM for a complete reaction with the maleic anhydride in mEVA were calculated to be 1.98 and 3.65 wt %, respectively. To optimize the reaction conditions, a range of FFA and BM concentrations were added between 0.5 and 1.5 times of 1.98 and 3.65 wt %, respectively. The obtained mEVA compounds were further compression-molded at 150 °C under 10 MPa for 20 min, with a 5 min preheat process and a 10 min cooling process, to obtain 1 mm thick films. The samples are denoted as DA*x* (or mEVA-DA*x*), where *x* is varied between 0.5 and 1.5, representing the concentrations of FFA and BM. The samples obtained were kept at room temperature for 2–3 days before analysis and shape memory characterization. In addition, the compounds without BM were prepared to analyze the reaction between FFA and maleic anhydride of mEVA; these samples are denoted as FFA*x* (or mEVA-FFA*x*), where *x* is varied from 0.5, 1 and 1.5 representing the concentrations of the FFA.

ZnMA, mEVA, and DCP were mixed at 80 °C with a rotor speed of 70 rpm to introduce ionic bonds to mEVA. The content of DCP was 0.5 wt % of mEVA, and the contents of ZnMA were 10 and 20 wt % of mEVA. The obtained compounds were compression-molded at 170 °C under 10 MPa for 20 min, with a 5 min preheat process and a 10 min cooling process, to obtain 1 mm thick films. These samples are denoted as ZnMA*x* (or mEVA-ZnMA*x*), where *x* is varied from 10 to 20, representing the contents of ZnMA. The samples obtained were used for structural analysis and mechanical and shape memory characterizations. In addition, self-polymerized ZnMA was prepared by reacting ZnMA and DCP (0.5 wt % of ZnMA) under the same conditions in order to obtain polyZnMA for control analysis. 

To introduce a dual network of DA and ionic bonds to mEVA, mEVA-DA 1 was further mixed with 10 wt % ZnMA and 0.5 wt % DCP in a mini-extruder under the same conditions as those of mEVA-ZnMA preparation. The obtained sample was named mEVA-DA1-ZnMA 10 wt %.

### 2.3. Characterization

The tensile testing and cyclic tensile experiments were performed using a Shimadzu Autograph AGS-X tester (Buckinghamshire, UK) equipped with an oven, with samples conforming to ASTM-D638-14 type V. For tensile testing, the extension rate was 50 mm min^−1^ with a 10 kN load cell, and the tests were carried out at room temperature according to ASTM-D638. During the cyclic tensile experiments, the specimen was firstly stretched to 200% strain and then unloaded to 0% strain to complete the first cycle. The specimen was then stretched again to 200% strain and unloaded to 0% strain to complete the second cycle. Then the specimen was relaxed at room temperature for 10 min before being further stretched to 200% strain and then unloaded to 0% strain. After the third cycle, the specimen was heated up to 130 °C and kept isothermally for 30 min before being cooled down to room temperature. The specimen was stretched to 200% strain and then unloaded to 0% strain to finish the fourth circle. All the stretching and unloading were conducted at 10 mm min^−1^. 

The shape memory behaviors of samples were analyzed using PerkinElmer DMA8000 (Buckinghamshire, UK). The experiments were conducted in stress control mode using Pyris software. Through DMA shape memory analysis, the percentages of shape fixity and shape recovery were determined. The samples analyzed were prepared by cutting the compression-molded films into strips of ~5 mm length, ~5 mm width, and ~1 mm thickness.

Fourier transform infrared spectra (FTIR) were collected using Bruker TENSOR 27 (Coventry, UK) at a resolution of 4 cm^−1^ with 32 scans. 

Scanning electron microscopy (SEM) images of the cryogenically fractured surfaces of the blends were carried out using a Zeiss SUPRA 55-VP field emission scanning electron microscopy system (Cambridge, UK). 

Dynamic mechanical thermal analysis (DMTA) was performed on a Tritec 2000 DMA (PerkinElmer, Buckinghamshire, UK) with 20 mm × 5 mm × 1 mm samples in tension mode from −120 to 250 °C using a temperature ramp of 3 °C·min^−1^, with 0.05 mm amplitude and a frequency of 1.0 Hz. 

Differential scanning calorimetry (DSC) experiments were conducted on a DSC 2910 (TA Instruments, Inc., Surrey, UK) with a heating–cooling–heating procedure. All measurements were carried out using a constant heating and cooling rate of 20 °C min^−1^ between 0 and 200 °C.

The crosslinking density was calculated based on an equilibrium swelling test. The samples (~0.5 g) were swollen in toluene at 25 °C for 72 h to achieve an equilibrium swelling state (see Figure S1, Supplementary Information). The weight of the samples was measured under the equilibrium swelling. Then, the samples were dried in a vacuum oven for 48 h at 50 °C to remove the solvent and were reweighed. The volume fraction of mEVA in the swollen gel, *V*_r_, can be used to represent the relative crosslinking degree and was determined by Equation (1):

(1) $$V_{r}=\frac{m_{0}\varphi \left(1-\alpha \right)\rho_{r}^{-1}}{m_{0}\varphi \left(1-\alpha \right)\rho_{r}^{-1}+\left(m_{1}-m_{2}\right)\rho_{s}^{-1}}$$

where *m*_0_ is the sample mass before swelling; *m*_1_ and *m*_2_ are the sample masses before and after drying, respectively, *φ* is the mass fraction of polymer in the sample, *α* is the mass loss of the polymer during swelling, *ρ*_r_ is the mEVA density (*ρ*_r_ = 0.962 g·cm^−3^), and *ρ*_s_ is the solvent density (toluene, *ρ*_s_ = 0.867 g·cm^−3^).

The elastically active network chain density, *v*_e_, which was used to represent the crosslink density, was then calculated by the Flory–Rehner Equation (2) [20]:

(2) $$\nu_{e}=-\frac{\ln\left(1-\nu_{r}\right)+\nu_{r}+\chi \nu_{r}^{2}}{V_{r}\left(\nu_{r}^{\frac{1}{3}}-\frac{\nu_{r}}{2}\right)}$$

where *v*_e_ is the crosslink density of the sample, *V*_s_ is the molar volume of the solvent (107 cm^3^ mol^−1^ for toluene), and *χ* is the Flory–Huggins interaction parameter between polymer and solvent. The *χ* between polymer and solvent is calculated from Equation (3) [20]:

(3) $$\chi =\frac{{\left(\delta_{s}-\delta_{r}\right)}^{2}V_{s}}{RT}$$

where *δ*_s_ and *δ*_r_ are the solubility parameters of the solvent (18.2 MPa^1/2^) and EVA, respectively, *R* is the universal gas constant, and *T* is the absolute temperature. The solubility parameter of EVA is calculated by Equation (4) [21]:

(4) $$\delta_{r}=\frac{\sum F_{i}}{\bar{v}}$$

where $\bar{v}$ is the molar volume of the repeating unit and *F*_i_ is molar attraction constant of the chemical group in the repeating unit.

## 3. Results and Discussion

### 3.1. Formation of Reversible Crosslinking Networks

**Figure 1:**
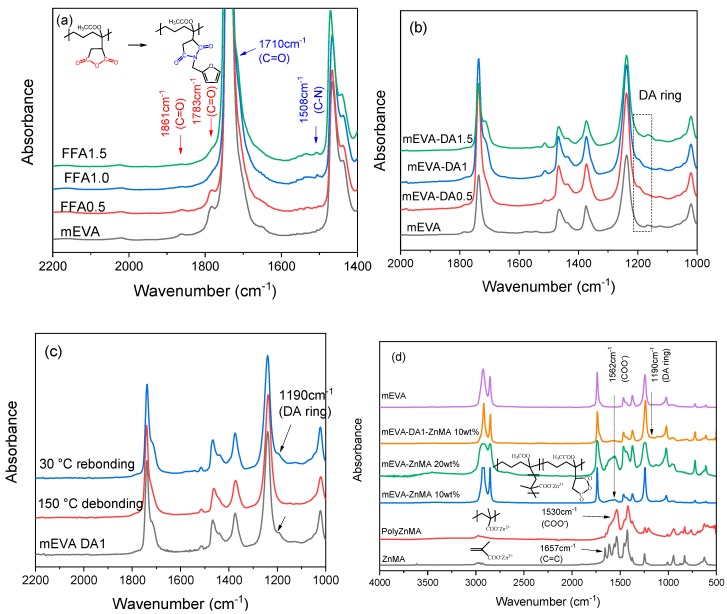
FTIR spectra: (**a**) furfurylamine grafting reaction onto mEVA; (**b**) Diels–Alder reaction between furfurylamine and 4,4’-methylenebis(N-phenylmaleimide); (**c**) crosslinked mEVA-DA; (**d**) ionic crosslinked mEVA by zinc methacrylate.

**Figure 2:**
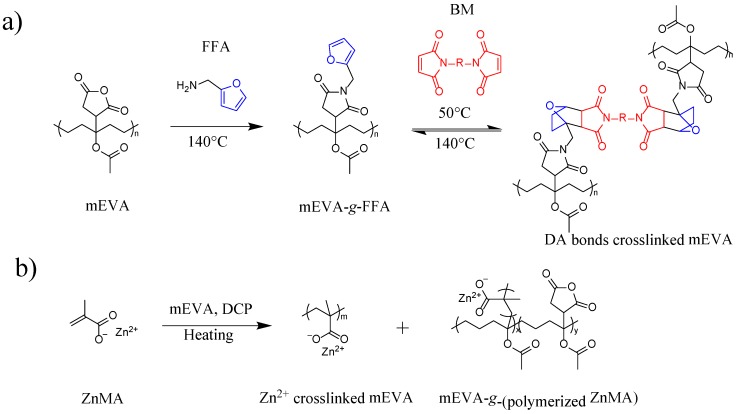
(**a**) Schematic of Diels–Alder crosslinking reaction in mEVA. (**b**) Introduction of Zn ions into mEVA.

To introduce reversible dynamic covalent bonds into mEVA, a typical DA reaction based on FFA and BM was conducted. As confirmed by FTIR in Figure 1a, the FFA reacted with the maleic anhydride (MA) group of mEVA and grafted onto the mEVA chains, causing the reduced intensity of the characteristic absorption peaks (1861 and 1783 cm^−1^) of MA. When the FFA content was equal to or greater than MA, the characteristic absorption peaks of MA completely disappeared. After addition of BM, the characteristic C–O stretch from the furan ring appeared at ~1190 cm^−1^, as shown in Figure 1b, indicating the occurrence of the DA reaction between FFA and BM, as illustrated in Figure 2. This resulted in dynamic DA crosslinking networks in mEVA. 

To evaluate the dynamic covalent DA network, the mEVA-DA*x* samples were firstly dissolved in THF at room temperature (see Figure S1). Whilst pristine mEVA fully dissolved, mEVA-DA samples only partially dissolved. The undissolved gel contents were calculated with Equation (1) to be 22, 62, and 57 wt % for mEVA-DA0.5, mEVA-DA1, and mEVA-DA1.5, respectively. The calculated crosslinking densities are 1.57 × 10^−4^, 2.36 × 10^−4^, and 1.71 × 10^−4^ mol/cm^3^ for mEVA-DA0.5, mEVA-DA1, and mEVA-DA1.5, respectively. The sample mEVA-DA1.5 exhibited a lower crosslinking density because the excess Diels–Alder bonding groups behaved as a plasticizer. 

To test the reversibility of the crosslinking structure of mEVA-DA, mEVA-DA1 was reheated to ~150 °C and kept isothermal for 10 min, and a decrease of the C–O absorption peak of DA ring at 1190 cm^−1^ was observed (Figure 1c). Subsequently, after the sample was cooled down to 30 °C and kept for 3 days at room temperature, the C–O stretch peak re-appeared. This demonstrates the reversibility of the covalent crosslinking structure in mEVA-DA samples. 

Next, by introducing ZnMA particles to mEVA-DA1, ionic bonds were constructed through the coordination reaction of ZnMA with the maleic anhydride group of mEVA, initiated by a trace amount of DCP during the melt-compounding process (see Figure 2b). ZnMA can react via two pathways. Firstly, it can graft onto the backbone of mEVA, forming ionic network structures. Secondly, it can self-polymerize into polyZnMA. The grafting of ZnMA onto the polymer backbone has been widely confirmed previously [13,22,23,24,25,26]. As characterized by FTIR shown in Figure 1d, the absorption peak for the C=C bonds at 1657 cm^−1^ of ZnMA disappeared after melt-mixing with mEVA. Additionally, the absorption peak of the carboxylate anion appeared at 1562 cm^−1^ in the mEVA-ZnMA blends, indicating a successful reaction. During the swelling tests, the crosslinking densities of crosslinked mEVA with 10 and 20 wt % ZnMA were calculated to be 2.73 × 10^−4^ and 7.22 × 10^−4^ mol/cm^3^, respectively. The introduction of 10 wt % ZnMA into mEVA-DA1 led to a crosslinking density of 3.74 × 10^−4^ mol/cm^3^, higher than that of mEVA-DA1, indicating an additional ionic crosslinking network. SEM imaging in Figure S2 demonstrates the morphological effect of the reversible Diels–Alder crosslinking and ZnMA ionic bonding on mEVA, demonstrating their interactions with the polymer and supporting the crosslinking density results.

### 3.2. Mechanical Performance of Reversibly Crosslinked mEVA

**Figure 3:**
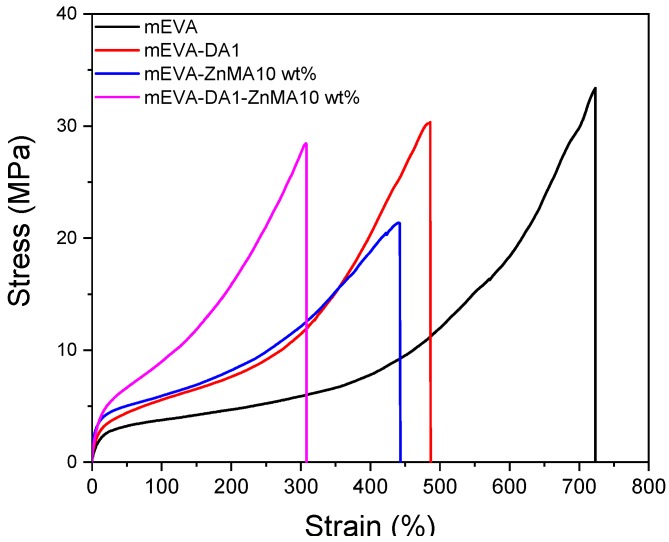
Tensile stress–strain curves of reversibly crosslinked mEVA by Diels–Alder reaction and ZnMA.

The mechanical properties of the crosslinked mEVA with dynamic bonds were characterized and are shown in Figure 3 and Table S1. The stress at 100% extension and Young’s modulus increased with the increase of dynamic covalent bonds and ionic interactions, especially for ZnMA-crosslinked mEVA. The DA-crosslinked mEVA showed higher elongation at break and tensile strength than the ZnMA-crosslinked mEVA due to the greater flexibility of DA crosslinking compared to ionic clusters. For pure mEVA and mEVA-DA1, mEVA-ZnMA 10 wt %, and mEVA-DA1-ZnMA 10 wt %, the tensile strengths were 33.4, 30.3, 21.4, and 28.4 MPa, respectively, the stress at 100% extension increased from 3.8 to 5.6, 5.9, and 9.0 MPa, respectively, and the elongation at break decreased from 723% to 486%, 441%, and 308%, respectively. The stress–strain curves for mEVA-DA0.5, mEVA-DA1.5, and mEVA-ZnMA 20 wt % are shown in Figure S3 and summarized in Table S1.

**Figure 4:**
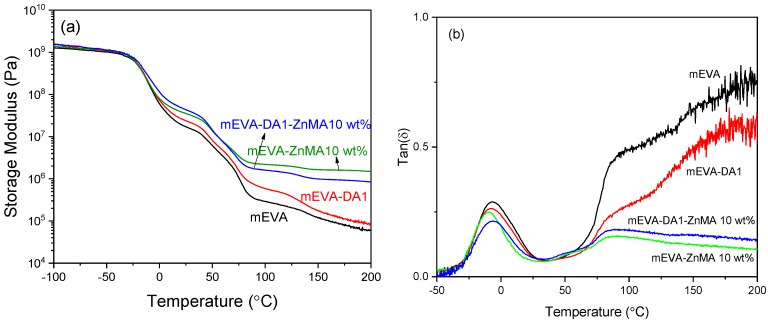
(**a**) Storage modulus–temperature curves and (**b**) tan *δ*–temperature curves of reversibly crosslinked mEVA by Diels–Alder reaction and zinc methacrylate during dynamic mechanical thermal analysis.

As expected, crosslinked mEVA had a higher storage modulus than pure mEVA at temperatures greater than −25 °C (Figure 4). Compared with pure mEVA and ZnMA-crosslinked mEVA, DA-crosslinked mEVA had a sharp decrease in storage modulus between 130–150 °C, attributed to the debonding of DA bonds. After the introduction of ZnMA into DA1-crosslinked mEVA, the modulus increased greatly, especially above ~70 °C, close to the melt temperature of mEVA, and no sharp decreases in storage modulus were observed between 130–150 °C, demonstrating the strength of the ionic interactions compared to the Diels–Alder bonding.

Additionally, the tan *δ* in Figure 4b shows that the *T_g_* for the polymers is largely unaffected by the incorporation of the Diels–Alder and ionic interactions, remaining at −5 °C. The large tan δ losses observed above 100 °C for mEVA and mEVA-DA1 are attributed to the melt of the polymer, which is not observed after the ionic interactions are introduced.

### 3.3. Shape Memory Behaviour of Reversibly Crosslinked mEVA

**Figure 5:**
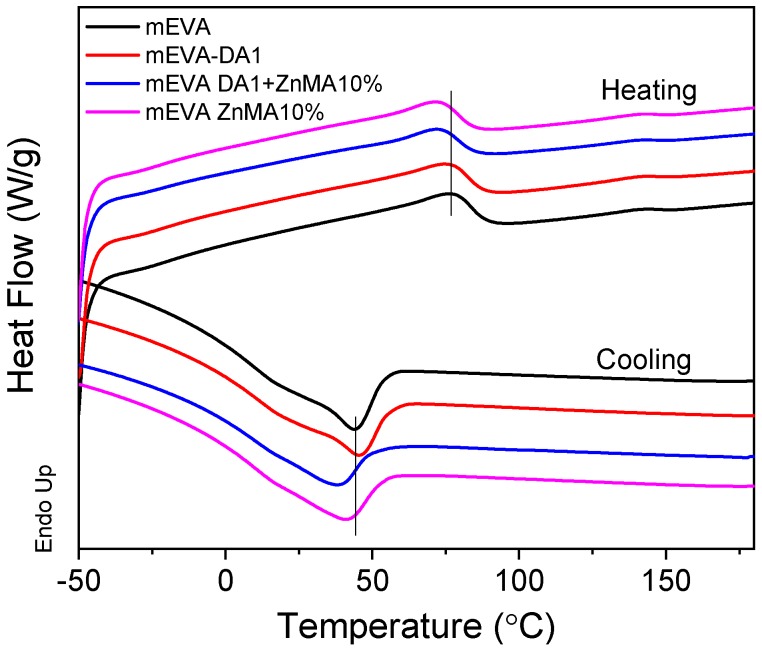
DSC scanning curves of reversibly crosslinked mEVA.

Shape memory effect of a polymer is a viscoelastic behavior involving the ability to fix a temporary shape followed by shape recovery. Under different external conditions, the dominant viscous or elastic behavior of the polymer promotes the transformation. Usually, the transition temperatures such as the glass transition temperature (*T*_g_), the melting temperature (*T*_m_), or the debonding temperature of reversible bonds can be used as the transition switch. At temperatures above *T*_g_, *T*_m_, or the debonding temperature, polymer chain segments possess good mobility, which enables changes in conformation; below *T*_g_, *T*_m_, or the debonding temperature, the chain segment motion is restricted, enabling the fixing of the temporary shape. The pure mEVA showed a melting temperature of 77 °C and a crystallization temperature of 44 °C (Figure 5), which can be used as the switches of shape recovery and shape fixing, respectively. However, the crystallization temperature of the composites are very close to room temperature, which is unfavorable to the shape fixing. Nonetheless, the addition of high-temperature dynamic crosslinking into mEVA assists in fixing the temporary conformation and additionally in the recovery ability due to the stronger chain networks. As shown in the DSC scanning curves, after mEVA was reversibly crosslinked, the melting temperature and the crystallization temperature of mEVA were both decreased up to 5–6 °C, resulting from the restrictions of crosslinking bonds on the movements of polymer chains. The first heating cycle is shown in Figure S4.

While investigating the shape memory effect of polymers, the deformation and recovery temperature is typically set as *T*_trans_ + 20 °C, and the fixing temperature is set as *T*_trans_ − 20 °C [27]. For reversibly crosslinked mEVA, there are three transition processes: melt–crystallization, DA debonding–rebonding, and ionic debonding–rebonding. Based on the DMA results discussed in Section 3.2 (Figure 4a), 130 °C was taken as the transition temperature for the deformation and recovery of the samples during shape memory tests. The DMA shape memory cyclic stress–temperature–strain tests were carried out in a tensile mode and consisted of four consecutive steps. Step 1 (programming) involves heating the samples to 130 °C at a heating rate of 5 °C/min. The samples were then kept in an isothermal state for 5 min (strain *ε*_i_) and then stretched until a strain of ~35% was reached (strain *ε*_s_). In steps 2 and 3 (shape fixing), the temporary shape was locked by cooling the samples to 30 °C, keeping the stress constant. The stress was then removed, and isothermal conditions were maintained for 5 min to allow the samples to adopt their temporary shape (strain *ε*_u_). In step 4 (recovery), the samples were heated to 130 °C at 5 °C/min and kept in an isothermal state for shape recovery to happen (strain *ε*_r_). The cycle was repeated two times, and the data were collected to plot three-dimensional shape memory graphs. The percentage of shape fixity and percentage of shape recovery were calculated based on Equations (5) and (6).

(5) $$\%\;Shape\;Fixity=\frac{\varepsilon_{u}-\varepsilon_{i}}{\varepsilon_{s}-\varepsilon_{i}}\times 100$$

(6) $$\%\;Shape\;Recovery=\frac{\varepsilon_{u}-\varepsilon_{r}}{\varepsilon_{u}-\varepsilon_{i}}\times 100$$

**Figure 6:**
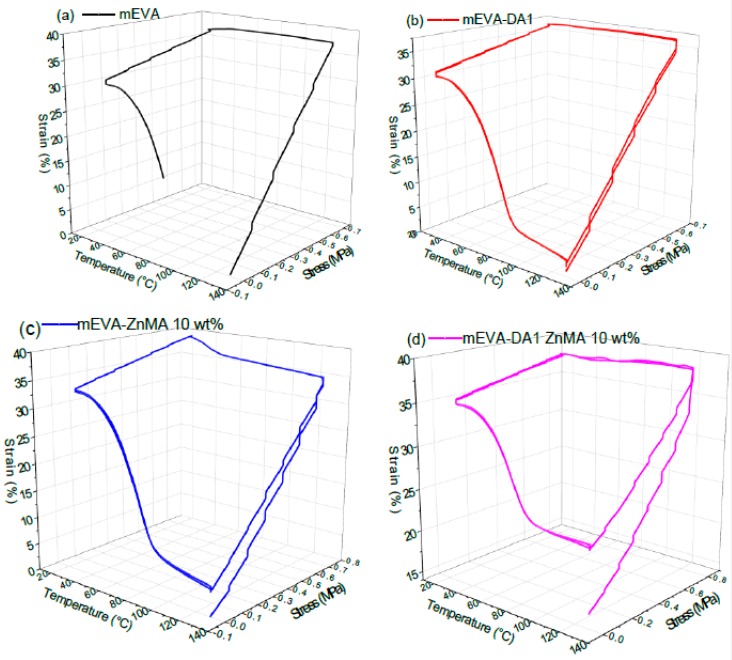
Three-dimensional shape memory stress–strain–temperature curves (two consecutive cycles): (**a**) mEVA; (**b**) mEVA-DA1; (**c**) mEVA-ZnMA 10 wt %; and (**d**) mEVA-DA1-ZnMA 10 wt %.

**Figure 7:**
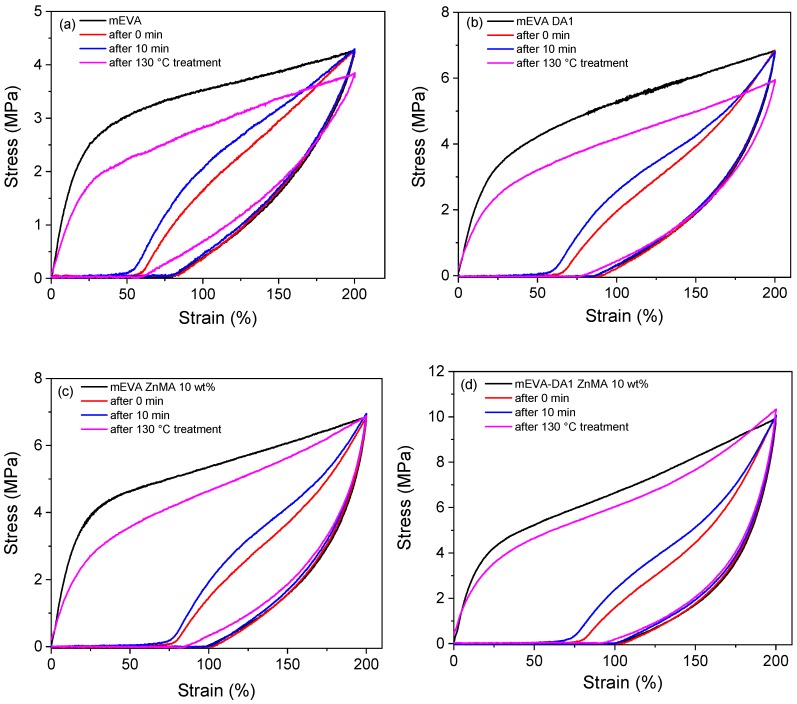
Cyclic tensile stress–strain curves of (**a**) mEVA, (**b**) mEVA-DA1, (**c**) mEVA-ZnMA 10 wt % and (**d**) mEVA-DA1-ZnMA 10 wt % of pristine materials, after 0 min recovery time and after 10 min recovery time at a 130 °C recovery temperature.

The shape memory effects of chemically crosslinked EVA using DCP and benzoyl peroxide (BPO) were studied and reported previously [28,29]. The shape memory cycles of mEVA and reversibly crosslinked mEVA samples are shown in Figure 6. A partial shape recovery was noticed in the first cycle, especially for samples with higher crosslinks: mEVA-ZnMA 10 wt % and mEVA-DA1-ZnMA 10 wt %. The hysteresis effect or incomplete recovery in the first cycle can be due to irreversible deformation of the reversibly crosslinked mEVA. The hysteresis effect has been studied and previously reported for polyurethane based shape memory polymers [30,31]. Irreversible morphological changes that involves reorganization of hard segments during the programming or stretching process for the first time in the reversibly crosslinked mEVA could be the reason for the incomplete recovery in samples c and d [32]. The percentages of shape fixity and shape recovery of mEVA and mEVA containing DA1, ZnMA 10 wt %, or DA1-ZnMA 10 wt % were calculated from the data collected from the second shape memory cycle, based on Equations (5) and (6), and are summarized in Table 1. Due to the hysteresis effect, the samples were not stretched to further ~35% strain in the second cycle and the percentages of shape fixity and recovery were calculated based on the maximum strain limit of the DMA instrument. The percentages of shape fixity of mEVA and mEVA containing DA1, ZnMA 10 wt %, DA1-ZnMA 10 wt % were 80.1%, 82.3%, 93.7%, and 76.4%, and the percentages of shape recovery were 51.5%, 95.6%, 99.0%, and 99.4%, respectively. For the mEVA sample, the shape recovery was measured at temperature close to the melting point (77 °C). Above the melting point (>77 °C), the mEVA sample melted and deformed without showing shape recovery. Even though there was hysteresis observed in the first cycle, after the introduction of DA and ionic crosslinks, the shape-recovery ability was greatly improved in the second cycle. It was noticed that the shape-fixing ability of crosslinked mEVA-DA1-ZnMA 10 wt % samples was reduced, and this was due to the significant reduction in crystallization, the main transition of shape fixing. From DSC cooling curves, the crystallization degree of mEVA with DA1-ZnMA 10 wt % was only 76% of that of pure mEVA. By contrast, better shape fixity (93.7%) was measured for the mEVA-ZnMA 10 wt % sample, and this could be due to increase of strain noticed (Figure 7c) in the cooling phase between 30 and 45 °C, which is the recrystallisation temperature of the sample on the basis of DSC experiment (Figure 5). The strain-increase effect, which is due to recrystallisation while shape fixing, was not very evident on mEVA, DA1, and DA1-ZnMA 10 wt % samples. The improvement of the recovery ability is mainly due to the reversible DA bonding and ionic crosslinking networks, which can be reformed at higher temperature and provide a recovering force. 

Cyclic tensile experiments were performed to study the fixing and recovery ratio of reversible crosslinked mEVA at a larger strain of 200%. Figure 7 shows the cyclic tensile stress–strain curves of reversibly crosslinked mEVA samples. After one loading–unloading cycle at room temperature, notable residual strains were observed without long fixing time, which were 78%, 84%, 96%, and 98% for mEVA, mEVA containing DA1, ZnMA 10 wt %, and DA1-ZnMA 10 wt %, respectively. Significantly larger hysteresis was observed for reversibly crosslinked mEVA, with the dissipating energy values of 4.78, 7.62, 8.30, and 10.6 MJ m^−3^ for mEVA, DA1, ZnMA 10 wt %, and DA1-ZnMA 10 wt %, respectively. Besides the elastic chain entanglements of mEVA, the energy dissipation related to the hysteresis originated from the breaking of the reversible bonds while loading to large strain. The residual strain decreased when the specimen was allowed to relax at room temperature without loading. The recovery in this process involves competition between the elasticity of the elastic chains and the strength of the temporarily re-formed reversible bonds [33]. Once the specimen was unloaded, the deformed internal structures (elastic chains, DA bonds, and ionic bonds) began to recover; therefore, the recovery efficiency increased after the introduction of hydrogen or ionic bonds, although the recovery rate was decreased at room temperature due to the increase in binding energy from DA bonds and especially from ionic bonds. 

After a short holding time (0 min) and a relatively long holding time (10 min) at room temperature, the residual strain recovery ratios for mEVA were 24% and 37%, respectively. These ratios were 29% and 52% for DA1, 28% and 46% for ZnMA 10 wt %, and 28% and 49% for DA1-ZnMA 10 wt %. After heat treatment at 130 °C for 30 min, the residual strains were 100% recovered for all samples. However, the dissipating energy did not completely recover, and the recovery ratios of the stress at 200% extension and the dissipating energy increased sequentially from mEVA to DA1 to ZnMA 10 wt % to DA1-ZnMA 10 wt %, which was different from the results of SBS/EMAA blends [34], in which the irreversible deformation at large strain was due to the introduction of the crystalline EMAA. For ZnMA 10 wt % and DA1-ZnMA 10 wt %, the stresses at 200% extension were completely recovered attributed to excellent recovery ability of these reversible crosslinks. However, for DA1, the stress at 200% extension was not completely recovered because of the slow rebonding rate of DA bonds. Table 2 summarizes the cyclic tensile behavior of reversibly crosslinked mEVA samples. At larger strain, the fixing ratio and recovery ratio were improved for the reversible crosslinked mEVA. 

## 4. Conclusions

This study has developed dual reversible crosslinked mEVA by introducing DA bonding and ionic bonding to mEVA through reactive melt processing. The dual reversible crosslinking networks in mEVA led to a crosslinking density of 3.74 × 10^−4^ mol·cm^−3^, with a tensile strength of 28.4 MPa and strain at break of 308%. The introduction of ionic bonding into DA-crosslinked mEVA increased the storage modulus without affecting its *T_g_*. The melting temperature and the crystallization temperature of the dual reversible crosslinked mEVA were decreased to 5–6 °C. The reversible crosslinks endowed the mEVA with excellent shape-recovery ability (>95%) without weakening the shape-fixing ability. A hysteresis effect or incomplete shape recovery was noticed for samples with greater crosslinking, i.e., mEVA-ZnMA 10 wt % and mEVA-DA1-ZnMA 10 wt % during the first cycle, which was due to reorganization of hard segments during the programming or stretching of samples. Shape recovery of 99.4% and shape fixity of 76.4% were observed for the dual reversible crosslinked mEVA-DA1-ZnMA 10 wt % from the second shape memory cycle. The introduction of dual reversible crosslinking networks to polymers through reactive melt processing could qualify the materials for industry-friendly, reprocessable, heat-shrinkable shape memory products for applications such as strain relief, electrical insulation, and mechanical and environmental protection.

## Figures and Tables

**Table 1:** The parameters in the shape memory testing of reversibly crosslinked mEVA calculated from the data collected from the second shape memory cycle, based on Equations (5) and (6).

	% Shape Recovery	% Shape Fixity
**mEVA**	51.5	80.1
**mEVA-DA1**	95.6	82.3
**mEVA-ZnMA 10 wt %**	99.0	93.7
**mEVA-DA1-ZnMA 10 wt %**	99.4	76.4

**Table 2:** The cyclic tensile behavior of reversibly crosslinked mEVA.

Sample	Dissipating Energy (MJ m^−3^)	Fixing Ratio (%)	Recovery Ratio (%)				
			Strain			Stress at 200% Extension	Dissipating Energy (MJ m^−3^)
	First Cycle	First Cycle	0 min	10 min	at 130 °C	at 130 °C	at 130 °C
mEVA	4.8	39	24	37	100	90	69
mEVA-DA 1	7.6	42	29	52	100	87	75
mEVA-ZnMA 10 wt %	8.3	48	28	46	100	104	80
mEVA-DA1-ZnMA 10 wt %	10.6	49	28	49	100	100	88

## Supplementary Materials

The following are available online at https://www.mdpi.com/2073-4360/12/4/983/s1, Supplementary Information containing images of the swelling testing, SEM images of the morphology of mEVA after the introduction of both Diels–Alder interactions and ionic crosslinking, stress–strain curves of mEVA-DA0.5, mEVA-DA1.5 and mEVA-ZnMA 20 wt % and DSC first heating scanning curves.
