# Supplementary material for: Coupling Dynamic Covalent Bonds and Ionic Crosslinking Network to Promote Shape Memory Properties of Ethylene-vinyl Acetate Copolymers
Source: Polymers (Basel). 2020 Apr 23;12(4):983. doi: 10.3390/polym12040983 (PMC7240482; doi:10.3390/polym12040983)
Supplement: Supplementary file 1 [file polymers-12-00983-s001.pdf]

Supporting Information

Coupling dynamic covalent bonds and ionic crosslinking network to promote shape memory properties of ethylene-vinyl acetate copolymers

Wenjing Wu,[1,2] Sreeni Narayana Kurup,[1] Christopher Ellingford,[1] Jie Li,[2] Chaoying Wan[1]*

[1]*International Institute for Nanocomposites Manufacturing (IINM), WMG, University of Warwick, CV4 7AL, UK*

[2]*Aerospace Research Institute of Materials & Processing Technology, 100076, Beijing, China*

**C.Wan, Chaoying.wan@warwick.ac.uk*

(A)

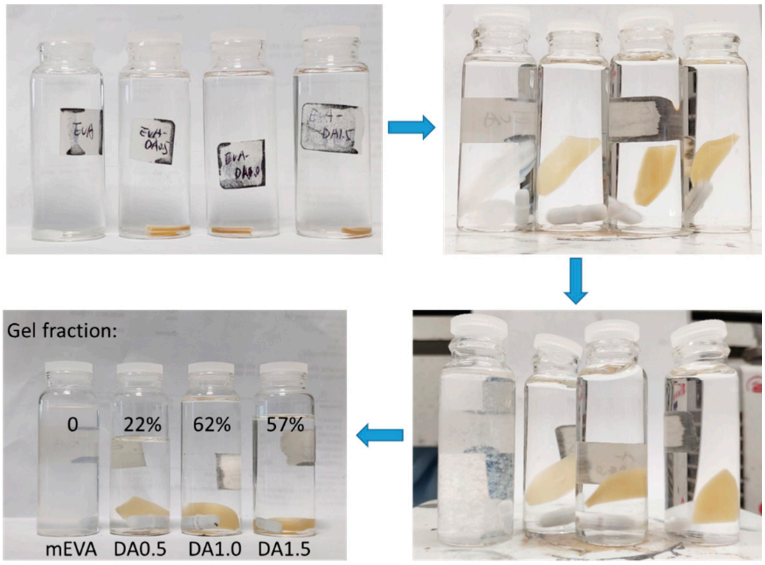

(B)

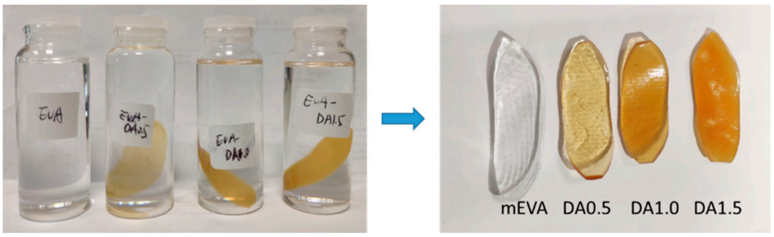

Figure S1 (A) The dissolving process of Diels-Alder reaction crosslinked mEVA in THF; (B) The swelling test of Diels-Alder reaction crosslinked mEVA in toluene.

Dispersion of ion clusters in mEVA

Figure S2 shows the morphologies of ZnMA particles and reversibly crosslinked mEVA. The pure ZnMA particles behaved a rod-like crystal morphology which changed to massive particles after the polymerization to polyZnMA (Figure S2c). After ZnMA was mixed into mEVA, the particles were dispersed as spherical particles of ~0.2 μm with clear boundary in the matrix before reaction (Figure S2d), and when the crosslinking reaction was finished in compression molding, the particles became much smaller and the boundary nearly disappeared (Figure S2e). For the mEVA crosslinked both DA and ZnMA (Fgiure S2f), the particle had a ellipsoidal shape and the boundary appeared again, which should be because the crosslinking points increased after further introduction of DA bonds and restricted the shearing dispersion of ZnMA particles.

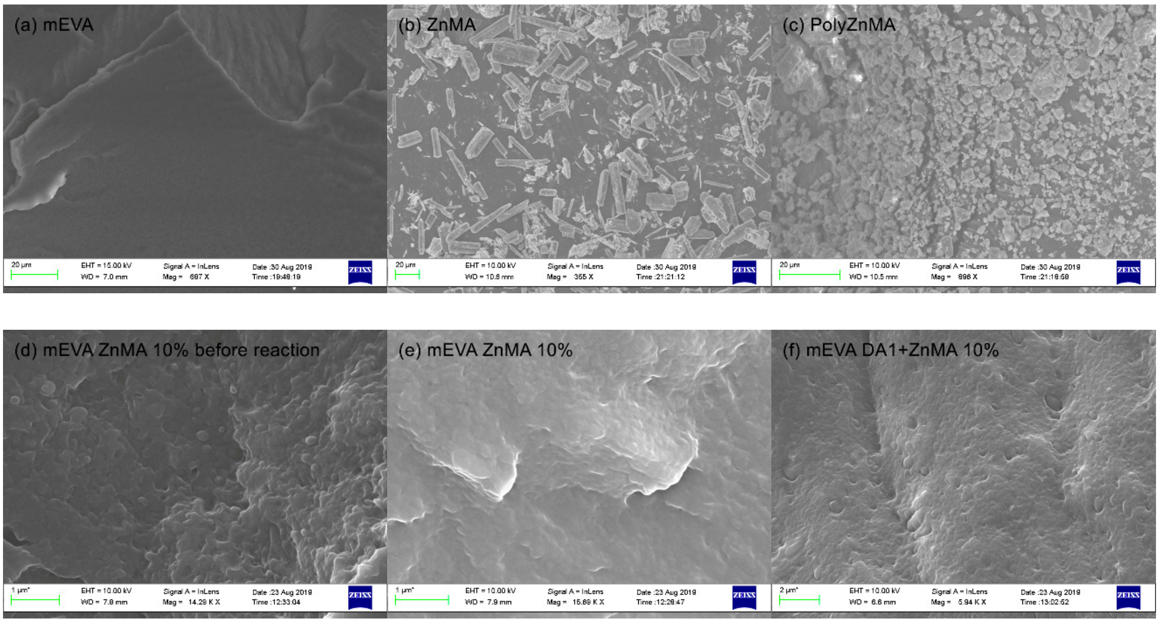

Figure S2. SEM images of (a) mEVA, (b) ZnMA, (c) PolyZnMA, (d) mEVA ZnMA 10% before reaction, (e) mEVA ZnMA 10%, (f) mEVA DA1 - ZnMA 10%.

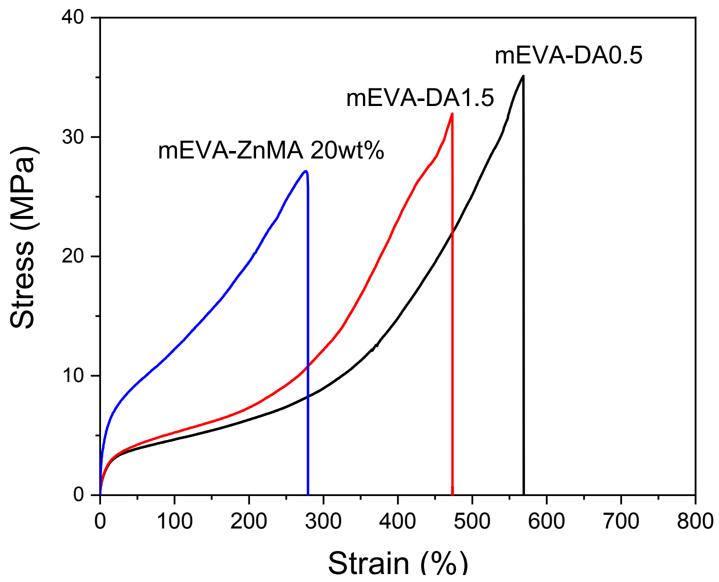

Figure S3. Stress strain curves of mEVA-DA0.5, mEVA-DA1.5 and mEVA-ZnMA 20 wt%.

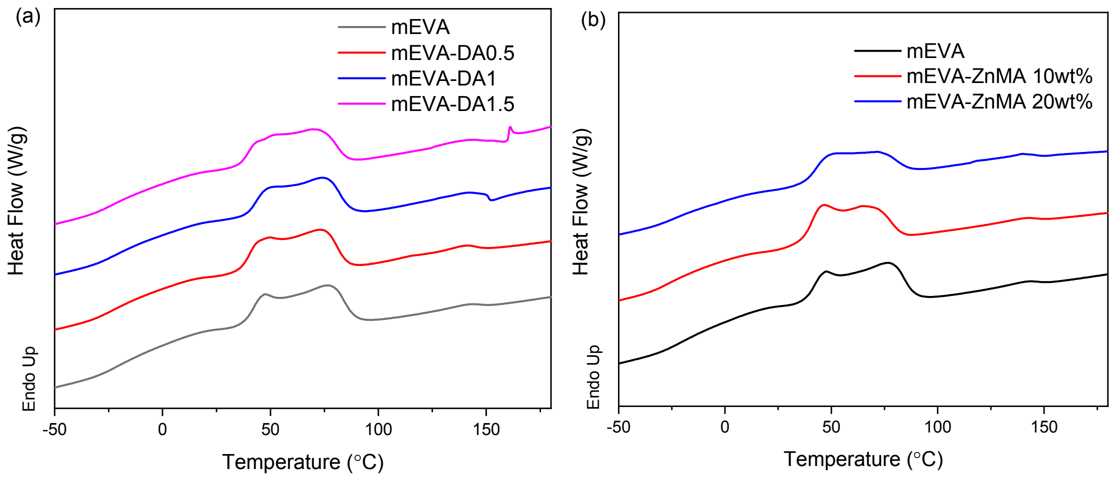

Figure S4. DSC first heating curves of reversibly crosslinked mEVA by Diels-Alder reaction (a) and zinc methacrylate (b).

Table S1. Summary of mechanical properties of reversibly crosslinked mEVA.

Sample	Young Modulus (MPa)	Stress at 100% extension (MPa)	Tensile Strength (MPa)	Elongation at break (%)
mEVA	58.5±3.2	3.8±1.1	33.4±3.5	723±46
mEVA-DA 0.5	86.4±9.8	4.7±0.9	35.1±3.9	569±45
mEVA-DA 1	120.3±13	5.6±1.2	30.3±4.1	486±68
mEVA-DA 1.5	147.0±27	5.2±0.8	32.0±3.5	473±63
mEVA-ZnMA 10wt%	247.1±35	5.9±0.9	21.4±2.8	440±57
mEVA- ZnMA 20wt%	340.0±23	12.2±1.1	27.1±2.4	276±42
mEVA-DA 1-ZnMA 10wt%	137.8±25	9.0±1.4	28.4±1.3	308±34
